# Surface‐Anisotropic Janus Silicon Quantum Dots via Masking on 2D Silicon Nanosheets

**DOI:** 10.1002/adma.202100288

**Published:** 2021-08-01

**Authors:** Marc Julian Kloberg, Haoyang Yu, Elisabeth Groß, Felix Eckmann, Tassilo M. F. Restle, Thomas F. Fässler, Jonathan G. C. Veinot, Bernhard Rieger

**Affiliations:** ^1^ WACKER‐Chair of Macromolecular Chemistry Catalysis Research Center Technical University of Munich Lichtenbergstraße 4 85758 Garching Germany; ^2^ Department of Chemistry University of Alberta Edmonton Alberta T6G 2G2 Canada; ^3^ Chair of Experimental Semiconductor Physics II Walter Schottky Institute and Physics Department Technical University of Munich Am Coulombwall 4 85748 Garching Germany; ^4^ Chair for Inorganic Chemistry with Focus on New Materials Department of Chemistry Technische Universität München Lichtenbergstraße 4 D‐85747 Garching Germany

**Keywords:** Janus particles, monolayer, silicane, silicon nanocrystals, silicon nanosheets, silicon quantum dots, surface anisotropic

## Abstract

Surface‐anisotropic nanoparticles represent a new class of materials that shows potential in a variety of applications, including self‐assembly, microelectronics, and biology. Here, the first synthesis of surface‐anisotropic silicon quantum dots (SiQDs), obtained through masking on 2D silicon nanosheets, is presented. SiQDs are deposited on the 2D substrate, thereby exposing only one side of the QDs, which is functionalized through well‐established hydrosilylation procedures. The UV‐sensitive masking substrate is removed through UV‐irradiation, which simultaneously initiates the hydrosilylation of a second substrate, thereby introducing a second functional group to the other side of the now free‐standing SiQDs. This renders surface‐anisotropic SiQDs that have two different functional groups on either side of the particle. This method can be used to introduce a variety of functional groups including hydrophilic and hydrophobic substrates, while the unique optoelectronic properties of the SiQDs remain unaffected. The anisotropic morphology of the QDs is confirmed through the aggregation behavior of amphiphilic Janus SiQDs at the interface of water and hexane. Additionally, anisotropic SiQDs are used to produce the first controlled (sub)monolayer of SiQDs on a gold wafer.

## Graphical abstract

Two‐faced “Janus” silicon quantum dots (QDs) are synthesized by using silicon nanosheets as a sacrificial mask. The method exploits the photosensitivity of the mask and the photoreactivity of the QDs to introduce two different functional groups on either side. The characteristic optoelectronic properties remain unaffected while the two‐sided nature imparts new unique properties such as self‐assembly and monolayer formation.

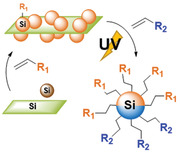

## 1 Introduction

Quantum dots (QDs) are semiconductor particles with dimensions smaller than the Bohr exciton radius in their parent bulk semiconductor.^[^ ^1^ ^]^ Their optoelectronic properties are dictated by quantum confinement and these size‐dependent characteristics have made them important active materials in displays, sensors, and biological applications, among others.^[^ ^2^ ^]^ Considering this broad material classification, silicon nanocrystals smaller than 4.5 nm in diameter are QDs. Silicon quantum dots (SiQDs) stand out from their compound semiconductor counterparts because they are toxic metal‐free and exhibit an indirect‐like band gap. SiQDs are particularly attractive because they are comprised of earth abundant elements, exhibit size‐dependent and chemically tunable photoluminescence (PL),^[^ ^3^ ^]^ are biocompatible,^[^ ^4^, ^5^ ^]^ and most notably offer a tailorable surface chemistry.^[^ ^6^, ^7^ ^]^ As such, it is possible to tune SiQD properties. Prototypical examples of this tunability include adjusting/controlling solubility,^[^ ^8^ ^]^ enhancing optoelectronic properties,^[^ ^7^ ^]^ and introducing enzymes or chromophores to impart specific biological activity;^[^ ^9^ ^]^ it is also possible to (self)‐assemble SiQDs into superstructures that offer the potential for heretofore unknown applications.^[^ ^10^, ^11^, ^12^ ^]^

In light of the crucial role surface chemistry plays in SiQD properties, seemingly countless reports have focused on the development of new functionalization methods.^[^ ^7^ ^]^ Without exception, these approaches have yielded isotropically functionalized nanoparticles (i.e., uniform functionalization across the particle surface). To our knowledge, there are no reports describing the preparation of anisotropically functionalized SiQDs (i.e., one side of the particle is modified with a different functional group than the other), also known as Janus particles.^[^ ^13^ ^]^ This conspicuous absence from the literature is certainly a reflection of the complexities associated with Si surface chemistry that is dominated by strong Si—C covalent bonding and is not amenable with ligand surface exchange that is commonly exploited in other functionalized nanoparticles. The two‐sided nature of Janus particles imparts unique properties such as surface activity,^[^ ^14^ ^]^ movement,^[^ ^15^ ^]^ and self‐assembly into superstructures, not accessible with isotropic counterparts.^[^ ^16^, ^17^ ^]^ In addition, Janus particles are promising materials for microelectronics,^[^ ^18^, ^19^ ^]^ coatings,^[^ ^20^ ^]^ and biological imaging,^[^ ^21^ ^]^ among many others.^[^ ^22^ ^]^ Clearly, realizing Janus SiQD particles would expand the scope of impact of these emerging materials.

The most straightforward approach to obtain Janus particles involves “masking” one side of the particles by temporarily immobilizing a monolayer of (nano)particles on a flat surface and modifying the exposed side.^[^ ^23^ ^]^ Removing the mask liberates the anisotropic particles and reveals a new surface on the particle that can be subsequently modified with a different functional group. A variety of masking substrates have been used including wax,^[^ ^24^ ^]^ glass,^[^ ^25^ ^]^ and polymers.^[^ ^26^ ^]^ However, these techniques suffer from low yields and/or are incompatible with nanoscale particles because of particle aggregation/agglomeration, rotation, and challenges associated with nanoparticle monolayer formation.^[^ ^22^, ^27^ ^]^ Unfortunately, methods for producing sub‐100 nm inorganic Janus particles remain limited.^[^ ^28^ ^]^ Using nanoscale surfaces (e.g., silica particles, single‐crystal polymers) as masks is a viable approach to overcome these limitations.^[^ ^29^, ^30^, ^31^ ^]^ In light of their large lateral size (i.e., microns), atomic scale thickness, and high specific surface area, solution‐dispersible 2D nanomaterials seem to be an ideal candidate for particle masking.^[^ ^32^ ^]^ However, a potential limitation is the subsequent mask removal, which is crucial to the entire process. Silicane, often referred to as silicon nanosheets (SiNSs), is photosensitive and decomposes cleanly under UV irradiation; its Si—H terminated surface also provides a convenient platform for nanoparticle attachment.^[^ ^33^ ^]^ Recently, SiQDs were directly attached to analogous 2D germanane through dehydrogenative coupling of silanes.^[^ ^34^ ^]^

Drawing inspiration from this knowledge, we report the first anisotropically functionalized SiQD Janus particles. We achieved this using silicane as a 2D substrate for the masking of SiQDs. These were deposited onto the silicane surface and functionalized using common hydrosilylation procedures. Subsequently, the sacrificial silicane mask was destroyed upon exposure to UV light and the liberated SiQDs were simultaneously functionalized via photo‐induced hydrosilylation.^[^ ^35^ ^]^ The resulting surface groups on the SiQDs were characterized and their anisotropic distribution on the particle surfaces was demonstrated through directed self‐assembly.

## 2 Results and Discussion

### 2.1 Janus SiQDs Synthesis

**Scheme 1:**
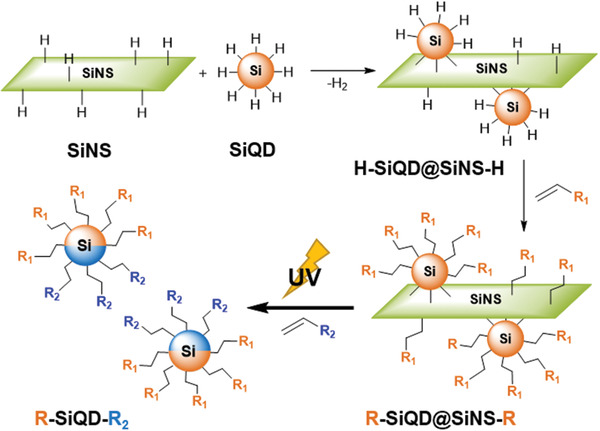
General synthetic pathway to obtain bifunctional Janus SiQDs. After deposition of SiQDs on SiNS and subsequent functionalization, the SiNS mask is removed through UV irradiation; simultaneously functionalizing SiQDs.

**Figure 1:**
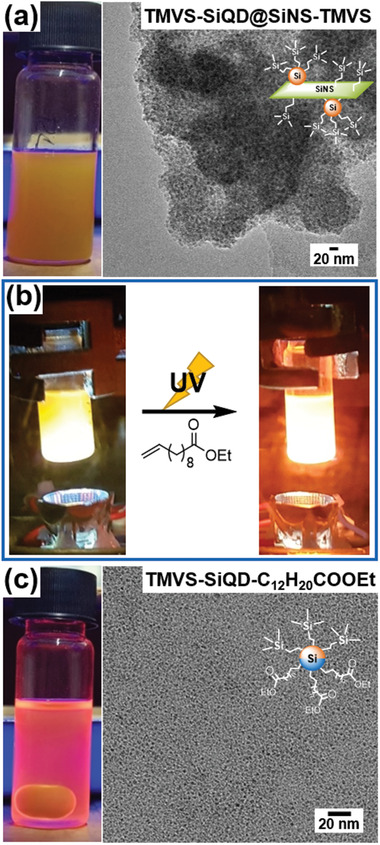
a) Picture of TMVS‐SiQD@SiNS‐TMVS (left) shows no PL response under UV‐light and TEM image confirms bonding of SiQDs to the SiNS surface (right). b) Pictures of the reaction of TMVS‐SiQD@SiNS‐TMVS with ethyl 10‐undecenoate in a UV reactor. After 5 min of exposure, the bright orange PL returns. c) Picture under UV light of the sample obtained after the UV reaction. The sample is less turbid and shows a strong PL response (left). TEM image of the sample confirms the removal of SiNSs (right).

Drawing on the work by Yu et al., we employed thermally induced dehydrogenative coupling to tether SiQDs to the surfaces of silicane (i.e., silicon nanosheets; SiNSs).^[^ ^34^ ^]^ Subsequently, trimethyl(vinyl)silane (TMVS) was introduced to the exposed surfaces of the SiNS‐bonded SiQDs using established hydrosilylation protocols (**Scheme** **1**). The resulting material (denoted TMVS‐SiQD@SiNS‐TMVS) did not render clear colloidal dispersions—as is usually the case for 3 nm SiQDs—and it no longer exhibited visible PL characteristic of the SiQDs (≈720 nm)^[^ ^3^ ^]^ or SiNSs (≈515 nm)^[^ ^33^ ^]^ (**Figure** **1**a, left). Transmission electron microscopy (TEM) images of TMVS‐SiQD@SiNS‐TMVS are consistent with SiQDs being bonded to the surface of the larger SiNSs (Figure 1a, right). Subsequent UV‐irradiation of TMVS‐SiQD@SiNS‐TMVS in the presence of ethyl 10‐undecenoate (C_10_H_19_COOEt) resulted in reemergence of visible PL after 5 min of exposure (Figure 1b) and clearing of the originally turbid suspension; characteristic of colloidal SiQDs (Figure 1c, left). These observations suggest the SiQDs (i.e., TMVS‐SiQD‐C_10_H_20_COOEt) were freed from the SiNS surface. Removal of insoluble byproducts, resulting from the decomposition of SiNSs, was achieved by centrifugation and/or filtration to provide pure Janus SiQDs (TMVS‐SiQD‐C_10_H_20_COOEt) and is confirmed by TEM (Figure 1c, right).

**Figure 2:**
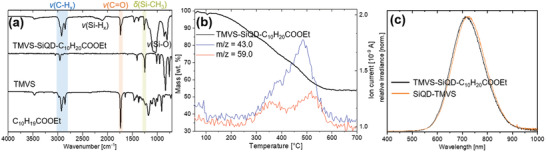
a) FT‐IR spectrum of TMVS‐SiQD‐C_10_H_20_COOEt with the corresponding starting substrates. b) TGA‐MS spectrum of the same sample and c) PL spectra of Janus SiQDs and a control sample obtained from the same synthesis.

The Fourier‐transform infrared (FT‐IR) spectrum obtained from the isolated TMVS‐SiQD‐C_10_H_20_COOEt reveals characteristic features arising from both target surface functionalities. We observe a band that is readily attributed to TMVS, δ(Si‐CH_3_) at 1260 cm^–1^, as well as a characteristic feature arising from C_10_H_19_COOEt; ν(C=O) at 1700 cm^–1^ (**Figure** **2**a). As expected, the Janus SiQDs also exhibit ν(C—H*_x_*) (≈2900 cm^−1^), δ(C—H*_x_*) (1460, 1360 cm^−1^), ν(Si—H*_x_*) (≈2100 cm^−1^), and ν(Si—O) (≈1000 cm^−1^) bands. Thermogravimetric analysis–mass spectrometry (TGA‐MS) of the identical sample further supports the successful attachment of the TMVS and C_10_H_20_COOEt functional groups (Figure 2b), as *m*/*z* ratios characteristic of the respective functional groups were detected; an *m*/*z*  =  43 arising from CO_2_ supports the presence of ester moieties, while an *m*/*z*  =  59 signal corresponds to dimethylsilane fragments, that fingerprint the TMVS functionality (TGA‐MS of comparative control samples are shown in Figure S1, Supporting Information). The FT‐IR spectrum of the isolated decomposition products revealed a strong ν(Si—O) band at ≈1000 cm^–1^, as well as spectral signatures of organic species at ≈2900 cm^−1^ (ν(C—H*_x_*)) (Figure S2). No further characterization of the byproducts was performed.

To corroborate the attachment of two different functional groups on the surfaces of the Janus SiQDs, chlorine and bromine were used as elemental markers for their qualitative detection in energy‐dispersive X‐ray spectroscopy (EDX). Indeed, when we repeated the procedure using 6‐chloro‐hex‐1‐ene and 11‐bromo‐undec‐1‐ene, subsequent EDX analyses showed chlorine and bromine in the final samples (Figures S3 and S4, Supporting Information). Unfortunately, the resolution of the EDX mapping precluded clear visualization of the localization of the functionalities, however both elements were co‐localized on the particles and show a homogenous distribution throughout the sample (Figures S3 and S4, Supporting Information). Bright field TEM imaging of the Janus SiQDs liberated from the silicane mask upon UV exposure showed no evidence of SiNSs (Figure 1c). The Janus QDs exhibit a size distribution of 3.40 ± 0.41 nm (Figure S5, Supporting Information) and solvodynamic radii of 3.26 ± 0.19 nm (Figure S6, Supporting Information). Consistent with SiQDs of this dimension, the PL maximum was noted at 720 nm upon excitation at 365 nm (Figure 2c).^[^ ^36^ ^]^ For comparison, an isotropically functionalized TMVS sample (SiQD‐TMVS) was synthesized in parallel from the identical SiQD/SiO_2_ composite and hydrofluoric acid (HF) acid liberation procedure. These isotropically functionalized SiQDs exhibit a statistically identical size (TEM: 3.38 ± 0.48 nm; dynamic light scattering (DLS): 3.11 ± 0.43 nm, Figures S6 and S7, Supporting Information) as well as the same PL emission maximum (Figure 2c). Calculated quantum yields (QY) of Janus and control SiQDs were found to be comparable with 31.7% and 26.9%, respectively. These results are consistent with the nature/properties of the SiQDs not being adversely impacted by the masking–demasking process.

### 2.2 Characterizing the Janus Nature of the Synthesized SiQDs

**Figure 3:**
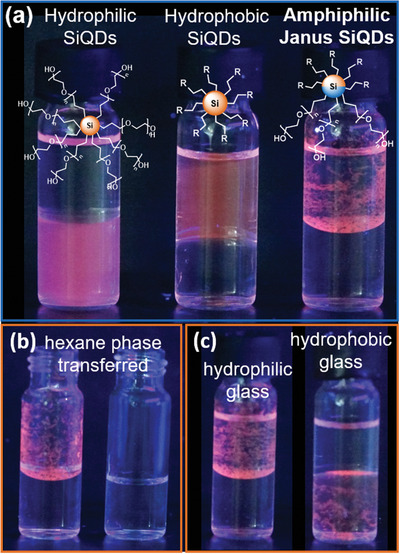
Pictures of SiQDs in a mixture of hexane and water under UV illumination. a) Self‐orientation of amphiphilic Janus SiQDs (right) causes arrangement at the interfaces of hexane/water and hexane/glass. The isotropic control samples (hydrophilic: left; hydrophobic: middle) partition into their preferred solvent. b) The hexane phase is transferred to a separate glass vial and shows no visible PL. c) Amphiphilic SiQDs are transferred to a hydrophobic glass vial causing arrangement at the water/glass (hydrophobic) interface.

Data presented thus far are consistent with the presence of two different functional groups on the SiQDs, however they do not differentiate between SiQDs where the two different moieties are randomly distributed on the surface and SiQDs with two chemically different “faces.” Microscopy is commonly used to show anisotropy in micrometer‐sized Janus particles, however, this is not possible with particles of nanoscale dimension; this is further complicated in the present investigation by the well‐established challenges of imaging SiQDs because of their low contrast. For sub‐100 nm Janus particles, many reports infer the particle anisotropy by examining their chemical behavior.^[^ ^37^ ^]^ In this context, we employed our SiNS masking procedure and prepared anisotropic SiQDs bearing hydrophobic (i.e., 1‐dodecene) and hydrophilic (i.e., allyloxy(polyethylene glycol)) faces. With these anisotropic amphiphilic SiQDs in hand, we examined their behavior in a water/hexane solvent mixture by visually monitoring their PL and compared it to that of identically sized isotropically functionalized hydrophobic (i.e., 1‐dodecene) and hydrophilic (i.e., allyloxy(polyethylene glycol) SiQDs. When exposed to the immiscible water/hexane mixture, amphiphilic SiQDs, prepared using the present masking procedure, preferentially reside at the water/hexane interface, but also localized at the interface of the hydrophilic glass vial and hydrophobic hexane phase (**Figure** **3**a right). In contrast, isotropically functionalized SiQDs partition into their respective solvents (Figure 3a left, middle). To address the issue of agglomeration of the amphiphilic Janus SiQDs at the hexane/glass interface, the hexane phase was carefully transferred to a separate vial. The SiQDs remain localized to the initial glass vial, while the transferred hexane phase does not show signs of PL (Figure 3b). In addition, when the amphiphilic Janus SiQDs are transferred to a glass vial that has been rendered hydrophobic, the SiQDs agglomerate at the interface of the hydrophilic water phase and the hydrophobic glass vial (Figure 3c). Videos of the transfer experiments are attached as Supporting Information. The behavior of the SiQDs prepared using our silicane masking procedure is consistent with that of other anisotropic amphiphilic particles and such behavior is commonly viewed as confirmation that an anisotropic Janus morphology has been achieved.^[^ ^14^, ^38^, ^39^, ^40^ ^]^

To further explore the behavior of Janus SiQDs, we examined and compared the self‐assembly behavior of anisotropic and isotropic SiQDs on gold wafers. Contact angle measurements and atomic force microscopy (AFM) images were used to gain insight on the exposed surface groups and deposition behavior. The synthetic strategy involved functionalizing SiQDs with chloro(dimethyl)vinyl silane (ClDMVS), which can subsequently react with a self‐assembled monolayer (SAM) of thioethanol on the gold surface (Figure S8, Supporting Information).^[^ ^41^ ^]^ This approach was chosen, because the direct synthesis of thiol‐capped SiQDs through hydrosilylation of alkenyl thiols is impossible due to the competing thiol–ene reaction. Accordingly, we synthesized anisotropic SiQDs bearing ClDMVS on one “face” and dodecyl groups on the other (H_25_C_12_‐SiQD‐ClDMVS), as well as isotropic control samples bearing only dodecyl groups (SiQD‐C_12_H_25_), only ClDMVS groups (SiQD‐ClDMVS) and statistically inserted ClDMVS and dodecyl groups (SiQD‐C_12_H_25_/ClDMVS). This “mixed” particle exhibits a virtually identical IR spectrum to the Janus SiQDs (Figure S9, Supporting Information); a demonstration of the ambiguity and challenges of verifying anisotropy. The thioethanol‐functionalized gold wafers are submerged in a solution of the SiQD samples overnight, upon which the wafers are washed and submerged in ethylene glycol, rendering any remaining ClDMVS groups hydrophilic. The ClDMVS bearing side of the Janus SiQDs (H_25_C_12_‐SiQD‐ClDMVS) is anticipated to preferentially interact with the thioethanol gold surface, thus exposing the dodecyl groups through their orientation. This behavior should lead to the gold surface exhibiting a hydrophobic contact angle (i.e., > 90°). By contrast, the isotropically functionalized particles are expected to exhibit a more hydrophilic contact angle for ClDMVS‐capped systems (SiQD‐ClDMVS, SiQD‐C_12_H_25_/ClDMVS) and no deposition should be observed for pure dodecyl SiQDs (SiQD‐C_12_H_25_).

**Figure 4:**
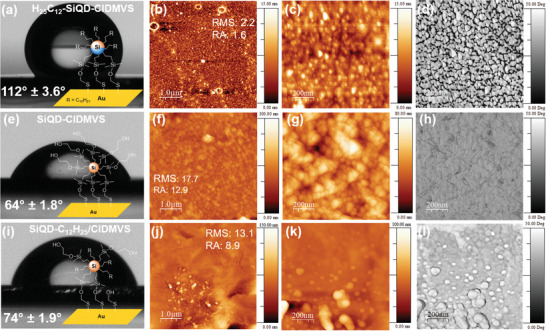
Immobilization of SiQDs on an SAM of thioethanol on gold wafers. Top row shows measurements of anisotropic SiQDs (H_25_C_12_‐SiQD‐ClDMVS) with a) contact angle, b,c) AFM micrographs of the gold wafer with roughness inset, and d) corresponding phase diagram. Middle row depicts e) contact angle of the control sample (SiQD‐ClDMVS) with f,g) AFM micrographs and h) phase diagram. Bottom row shows the i) contact angle of the control (SiQD‐C_12_H_25_/ClDMVS) as well as j,k) AFM micrographs with the corresponding l) phase image.

The reference thioethanol wafer exhibits a contact angle of 55° (Figure S10a, Supporting Information). Deposition of the isotropic SiQD‐ClDMVS and subsequent treatment with ethylene glycol result in an expected hydrophilic contact angle of 64° (**Figure** **4**e). For SiQD‐C_12_H_25_, we observe a contact angle of 78° (Figure S10e, Supporting Information). Here, no change in contact angle was predicted, however, a contact angle of 78° is still too hydrophilic to suggest SiQD‐C_12_H_25_ deposition. The measured contact angle of 74° for the isotropic mixed SiQD‐C_12_H_25_/ClDMVS sample seems reasonable, since both hydrophilic and hydrophobic groups are present on the surface (Figure 4i). Decisively, the Janus SiQD sample exhibits a contact angle of 112° suggesting a very hydrophobic surface on the gold wafer (Figure 4a). This value is consistent with literature‐reported values of dodecyl surfaces, implying the presence of a dodecyl‐(mono)layer, which is only possible if the SiQDs are anisotropic and show preferential orientation.^[^ ^42^, ^43^ ^]^

The contact angle results are corroborated by AFM measurements of the gold wafers, wherein the Janus sample (H_25_C_12_‐SiQD‐ClDMVS) exhibits a thin layer (up to 10 nm) of SiQDs with a slight increase in surface roughness (Figure 4b) compared to the reference thioethanol gold wafer (Figure S10b, Supporting Information). Large circular agglomerations are also visible, which are likely micellar structures that formed during the drying process—another indication of self‐assembly behavior. At larger magnification, distinct features around the regular grain structure can be observed (Figure 4c), which correspond to a significantly higher change in phase space (Figure 4d), especially compared to the phase changes in the reference samples (Figure 4h,l). These features indicate a sudden change in interaction forces caused by the hydrophobicity of the exposed side of the SiQDs and the hydrophilicity of the underlying substrate. This is indicative of a (sub‐)monolayer of SiQDs with a partially exposed hydrophilic substrate (thioethanol gold wafer) showing through; past the grain structure of the SiQDs.

The micrographs of the control samples do not exhibit this behavior. SiQD‐C_12_H_25_ shows no indication of deposition, as the surface roughness remains within the margin of error to the original thioethanol wafer (Figure S10, Supporting Information). Both isotropic control samples, SiQD‐ClDMVS and SiQD‐C_12_H_25_/ClDMVS, show a thick layer of SiQDs (up to 80 nm) with high surface roughnesses (Figure 4f,j). A homogenous distribution of large agglomerations with heights above 100 nm are visible in the SiQD‐ClDMVS sample (Figure 4g), while SiQD‐C_12_H_25_/ClDMVS is much more inhomogenous, likely caused by the random distribution of the two functional groups on the SiQD surface (Figure 4k). Only the Janus SiQDs are able to controllably orient and deposit themselves onto the gold wafer, resulting in the formation of a SiQD monolayer (Figure 4d). The evidence provided by the agglomeration behavior of the SiQDs at the interface of water and hexane, and by the observations made with the self‐assembly on gold wafers, leads to the conclusion that the synthesis procedure using silicane as a mask indeed results in the anisotropic functionalization of SiQDs, rendering anisotropic particles with two chemically different sides, also known as Janus particles.

## 3 Conclusion

We have demonstrated the use of a 2D nanomaterial, silicane, as a solid‐state masking substrate for the anisotropic modification of SiQDs. Since the deposition onto silicane is not specifically limited to SiQDs, we anticipate that this method forms the foundation for a broad scope of future investigations, including the application to other nanoparticles. Here, we use the dehydrogenative coupling of silanes to deposit SiQDs onto the surface of the 2D silicon nanomaterial. The exposed Si—H groups of the SiQDs are subsequently functionalized with common hydrosilylation techniques. The resulting material is irradiated with UV light in the presence of another alkene, which results in the decomposition of the 2D mask and simultaneous hydrosilylation with the newly exposed side of the SiQDs, yielding anisotropic Janus nanoparticles. The resulting SiQDs were characterized and the anisotropic morphology is confirmed through the arrangement of amphiphilic Janus SiQDs at the interface of water and hexane. The quality of the SiQDs remains unchanged throughout the procedure, exemplified by the comparison of size‐distributions and PL of the Janus QDs with isotropic control samples. This versatile procedure enables the synthesis of a vast library of unprecedented materials, combining the unique properties of SiQDs with the unique properties of Janus particles. Conceivable applications include the use as building blocks for large hierarchical superstructures or as biological mimics in fluorescent imaging. We have shown that this procedure enables the anisotropic hydrosilylation of a variety of functional groups, with the attachment of chloro(dimethyl)vinyl silane providing a platform for post‐synthesis modification. This way we were able to controllably immobilize a monolayer of Janus SiQDs onto the surface of a thioethanol‐coated gold wafer, on the one hand demonstrating the unique self‐assembly behavior of anisotropic nanoparticles and on the other, preparing the first monolayer of SiQDs, enabling future, in‐depth, fundamental characterizations of isolated, single SiQDs.

## 4 Experimental Section

### General Information

All reactions were performed under Schlenk conditions. The reactants and solvents were purchased from Sigma‐Aldrich unless stated otherwise, dried with molecular sieves (4 Å) and degassed by sparging with Ar for at least 20 min. Acetone was dried by passing over a column of heat‐dried basic silica. Allyloxy(polyethylene glycol) (Gelest) was first heated to 50 °C in a vacuum oven overnight prior to storage over molecular sieves. For water/hexane agglomeration experiments, glass vials were rendered hydrophobic by adding two drops of hexamethyldisilazane (HMDS) and heating to high temperatures using a hot air gun. Residual HMDS was removed in vacuum.

### Measurement Information

FT‐IR spectra were measured with a Vertex 70 FT‐IR using a Platinum ATR from Bruker.

The scanning electron microscope (SEM) images were obtained on a field emission (FE)‐SEM JSM 7500F from Jeol at an accelerating voltage of 1 kV. An INCA system by Oxford Instruments with an accelerating voltage of 10 kV was used for element mapping and EDX measurements.

The PL spectra were measured in an appropriate solvent with an AVA‐Spec 2048 from Avantes using a Prizmatix (light‐emitting diode (LED)) light source.

The quantum yield was determined by using a relative method using rhodamine B in ethanol as a standard (QY_R_  =  68%).^[^ ^44^, ^45^, ^46^ ^]^ See the Supporting Information for calculations (Figure S12, Supporting Information). UV/Vis measurement for absorbance values were carried out on a Varian Cary 50.

The DLS measurements were performed using a Malvern Zetasizer Nano ZS with a laser wavelength of 633 nm. Each measurement was consisted of 20 acquisitions with an acquisition time of 20 s.

The TGA‐MS measurements were conducted in a glove box on a Netzsch TG 209 F 1 Libra coupled with a QMS 403 Aëolos Quadro mass spectrometer by first annealing the sample at 100 °C for 20 min and ramping to a final temperature of 700 °C rate at a rate of 10 K min^−1^.

Contact angle measurements were performed on a Krüss DSA25 goniometer by averaging the results of five droplets (volume of deionized water droplet was 5 µL).

Bright field TEM images were taken with a JEOL JEM‐ARM200CF S/TEM electron microscope at an accelerating voltage of 200 kV. TEM samples were prepared by depositing a droplet of diluted suspensions in toluene onto a holey carbon‐coated copper grid (obtained from Electron Microscopy Inc.). The grid was kept in a vacuum chamber for at least 24 h prior to data collection.

TEM images for size distributions were obtained with a Ruby CCD camera on a JEM 1400 plus microscope by Jeol with an accelerating voltage of 120 kV. Size distributions were determined by measuring a minimum of 200 nanoparticles using ImageJ software.

AFM was measured in tapping mode with a Bruker Multimode AFM using NSG30 tips by scanning over 5 × 5 or 1 × 1 µm areas.

*Powder X‐ray diffraction*: For powder X‐ray diffraction (PXRD) measurements, the samples were ground in an agate mortar and sealed inside 0.5 mm glass capillaries. PXRD measurements were performed at room temperature on a STOE Stadi P diffractometer equipped with a Ge(111) monochromator for Mo Kα1 radiation (λ = 0.7093 Å), and a Dectris MYTHEN DCS 1K solid‐state detector.

### Synthesis of 4‐Decyldiazobenzene tetrafluoroborate (4‐DDB)^[^ ^41^ ^]^

4‐decylaniline (1.17 g, 5 mmol, 1 eq.) was added to a mixture of acetic acid (9 mL), propionic acid (9 mL), and HBF_4_ (50 wt%, 6 mL). After the mixture was cooled to 0 °C, NaNO_2_ (0.52 g, 7.5 mmol, 1.5 eq.) was slowly added over 30 min and the suspension was stirred for an additional 30 min. The suspension was precipitated in ice water, the red solid was filtered off, washed with ice water, and dried in a reduced atmosphere. The red product, 4‐decyldiazobenzene tetrafluoroborate (1.33 g, 4 mmol, 82%), was stored in a fridge.

^1^H NMR (400 MHz, CHCl_3_‐*d, δ*): 8.56–8.50 (m, 2 H), 7.61 (m, 2H), 2.86–2.77 (m, 2 H), 1.72–1.58 (m, 2 H), 1.35–1.18 (m, 14 H), 0.85 (m, 3 H).

### Synthesis of CaSi_2_

CaSi_2_ was synthesized according to the literature via melting of calcium (Alfa Aeser, 99.5%) and silicon (Wacker, 99.99%) in an electric arc furnace.^[^ ^47^ ^]^ Inside a glove box (MBraun, *p*(H_2_O), *p*(O_2_) < 0.1 ppm), stoichiometric amounts of calcium (426.4 mg, 10.6 mmol, 1 eq.) and silicon (594.3 mg, 21.2 mmol, 2 eq.) were mixed together and pressed into a pellet. The pellet was melted in an electric arc furnace (Edmund Bühler MAM1) placed in the glove box. The resulting regulus was grounded in an agate‐mortar, pressed to a pellet, and melted again in the arc furnace. A silver‐colored metallic regulus was obtained yielding phase pure CaSi_2_ as confirmed by PXRD (see Figure S11, Supporting Information). For further use, the CaSi_2_ regulus was grounded using an agate‐mortar.

### Synthesis of Silicane

The synthesis followed known literature procedures.^[^ ^47^, ^48^ ^]^

A Schlenk flask charged with HCl (100 mL, 37% aq.) was cooled to −30 °C and CaSi_2_ (1 g) was added. After 7 days of exfoliation, the yellow‐colored flakes were filtered in a Schlenk‐frit and washed with dry and degassed acetone (3 × 25 mL) to remove any remaining CaCl_2_. The flakes were dried under vacuum and stored in an Ar‐filled glovebox.

### Etching of Silicane with HF^[^ ^47^ ^]^

Silicane (SiNS, 40 mg) was dispersed in dry and degassed ethanol (2 mL) and ultrasonicated for 5 min to break up large agglomerates. The dispersion was transferred to a fluorinated ethylene propylene (FEP) container and water (1 mL) and HF (0.25 mL, 48% aq.) were added. Immediately, the etched silicanes (SiNS‐H) were extracted with dichloromethane (3 × 5 mL) and transferred to an FEP centrifuge tube. The tube was filled with toluene (to facilitate centrifugation) and centrifuged at 9000 rpm for 5 min. The supernatant was discarded and the residue (SiNS‐H) was re‐dispersed in dry acetone (≈10 mL). After centrifugation (9000 rpm, 5 min), the residue was dispersed in dry toluene (≈10 mL). The final centrifugation (9000 rpm, 5 min) yielded hydride‐terminated silicanes (SiNS‐H) that could immediately be used for further reactions or freeze‐dried from dry benzene for storage and later use. Around 30 mg was obtained from etching 40 mg silicane.

### Synthesis of SiQD/SiO_2_ Composite

A detailed description of the synthesis of the 3 nm SiQD in SiO_2_ composite was found in the literature.^[^ ^6^ ^]^ Hydrogen silsesquioxane (7.0 g) was put into a quartz boat and placed in an oven (Nabertherm RD 30/200/11). A steady stream of H_2_ in Ar (5%/95%) was applied and the oven was heated to 1100 °C at 4 °C min^−1^. The peak temperature was maintained for 1 h. After cooling to room temperature, the solid was ground to a fine powder using an agate mortar and pestle and subsequently shaken with high purity silica beads in a WAB Turbula mixer overnight. The final SiQD/SiO_2_ composite was then be etched using HF acid.

### Liberation of SiQD‐H from SiQD/SiO_2_ Composite with HF

In a polypropylene beaker charged with a Teflon‐coated stir bar, per 100 mg of SiQD/SiO_2_ composite, 1 mL ethanol, 1 mL water, and 1 mL HF acid (48% aq.) was added and the dispersion was stirred for 45 min. The etched silicon quantum dots (SiQD‐H) were extracted with toluene (3 × 10 mL) and centrifuged (9000 rpm; 5 min). The supernatant was discarded, and the residue was washed and centrifuged once with dry acetone (≈15 mL) and once with dry toluene (≈15 mL). The resulting SiQD‐H was used directly for reactions or freeze‐dried from benzene for storage in a glove box. Typically, per 100 mg of etched SiQD/SiO_2_ composite, 2 mg of SiQD‐H was obtained.

### General Procedure for the Hydrosilylation of a Given Alkene with SiQDs^[^ ^49^ ^]^

SiQD‐H (4 mg, obtained from etching 200 mg SiQD/SiO_2_) was dispersed in dry toluene (2 mL). 2 mmol of alkene and 4‐decyldiazobenzene tetrafluoroborate (6 mg) was added. The dispersion was degassed via three freeze–pump–thaw cycles and stirred for 16 h at room temperature. The dispersion was precipitated in an antisolvent (10 mL) and centrifuged (9000 rpm, 5 min). It was noted that the solvent/antisolvent mix must be able to dissolve the alkene. The residue was dispersed in a minimal amount of solvent (0.5 mL), antisolvent was added (10 mL), and the dispersion was centrifuged. This cycle was repeated an additional two times to yield functionalized silicon quantum dots (SiQD‐R).

### Synthesis of SiQDs Deposited on SiNSs and Subsequent Functionalization (R‐SiQD@SiNS‐R)

The procedure used an adapted literature‐known method to deposit silicon nanocrystals (SiQD‐H) on silicane (SiNS‐H).^[^ ^34^ ^]^ The subsequent hydrosilylations use established methods to modify the SiQDs’ surface with desired alkenes.^[^ ^35^, ^49^ ^]^

SiQD‐H (20 mg, obtained from etching 1 g of SiQD/SiO_2_) was dispersed in dry toluene (5 mL) and transferred to a heat‐dried Schlenk tube. SiNS‐H (30 mg, obtained from etching 40 mg silicane) was dispersed in dry toluene (2 mL) and added to the tube. The dispersion was degassed in three freeze–pump–thaw cycles and stirred at 100 °C for 2 days (the resulting dispersion could be centrifuged under Ar and freeze‐dried from benzene for storage).

The resulting dispersion was cooled to room temperature and a desired alkene (3 mmol) was added. After the addition of 4‐decyldiazobenzene tetrafluoroborate (10 mg), the dispersion was subjected to another three freeze–pump–thaw cycles and stirred for 16 h at room temperature. The dispersion was then centrifuged (9000 rpm, 5 min) directly in the reaction solvent. The supernatant might exhibit PL and should contain any isotropic SiQDs‐R that did not react with the SiNS‐H in the first step. The nonluminescent residue contained the desired R‐SiQD@SiNS‐R and was washed and centrifuged (9000 rpm, 5 min) three times with a solvent that could dissolve the respective isotropic SiQD‐R. For example, if the desired alkene was trimethyl(vinyl)silane, the residue was washed in toluene. The final R‐SiQD@SiNS‐R was freeze‐dried from benzene (for characterization) or used directly for the next step.

### Synthesis of Janus SiQDs from R‐SiQD@SiNS‐R

R‐SiQD@SiNS‐R was dispersed in dry toluene (or solvent of choice; neat alkene was also possible) and transferred to a heat‐dried Schlenk‐tube. Desired alkene (2 mmol) was added and the dispersion was degassed via three freeze–pump–thaw cycles. The functionalization was carried out using a near‐UV LED light source (360 nm). The light source was consisted of a single LED operating at 3 W mounted on a water‐cooled metal plate that was put on top of a magnetic stirrer. The Schlenk‐tube was mounted over the LED and the dispersion was irradiated and stirred for 8 h. The dispersion showed an intense PL after 5 min of irradiation. After completion of the reaction, the dispersion was centrifuged at 3000 rpm for 5 min. The residue (light brown to white) contained the byproducts of silicane's UV‐degradation while the supernatant contained the Janus SiQDs (showed PL). The supernatant was separated, and the residue was washed and centrifuged. The supernatants were combined and reduced in vacuum to facilitate subsequent precipitation. The combined supernatants were precipitated in an antisolvent (e.g., in the case of ethyl undecenoate: in methanol) and centrifuged (9000 rpm; 5 min). The precipitate was re‐dispersed in solvent and precipitated with an antisolvent. This re‐dispersion, precipitation, centrifugation cycle was repeated two times to yield purified Janus SiQDs (R‐SiQD‐R_2_).

### Self‐Assembly of SiQDs on Thioethanol‐Functionalized Gold Wafers

Preparation of the gold wafers was achieved through thermal evaporation of a 3 nm thick titanium layer followed by 50 nm of gold onto a silicon 〈100〉 wafer at a speed of roughly 1 Å s^−1^. The base pressure before evaporation was better than 10^−6^ mbar. The wafer was subsequently cleaved into 5 × 5 mm pieces and cleaned by ultrasonication in water, isopropanol, and acetone; each for 5 min. The sample was submerged in a 2 m solution of thioethanol in ethanol for 16 h. Afterward, it was washed with dry ethanol and dried using an Ar stream to yield a gold wafer with an SAM of thioethanol (water contact angle 55°).

SiQDs (3 mg) were dispersed in dry toluene (2 mL) and the gold wafer was submerged in the dispersion for 16 h. The wafer was carefully washed with dry toluene, dried with an Ar stream, and submerged in dry ethylene glycol for 16 h. The gold wafer was washed with ethylene glycol and dried under an Ar stream.

## Conflict of Interest

The authors declare no conflict of interest.

## Supporting information

Supporting Information

Supplemental Video 1

Supplemental Video 2

## Data Availability

The data that support the findings of this work are available in the Supporting Information of this article.
