# Supplementary material for: Surface‐Anisotropic Janus Silicon Quantum Dots via Masking on 2D Silicon Nanosheets
Source: Adv Mater. 2021 Aug 1;33(38):2100288. doi: 10.1002/adma.202100288 (PMC11468500; doi:10.1002/adma.202100288)
Supplement: Supplementary file 1 — Supporting Information [file ADMA-33-2100288-s002.pdf]

© 2021 Wiley-VCH GmbH

ADVANCED MATERIALS

Supporting Information

for *Adv. Mater.,* DOI: 10.1002/adma.202100288

Surface-Anisotropic Janus Silicon Quantum Dots via Masking on 2D Silicon Nanosheets

Marc Julian Kloberg, Haoyang Yu, Elisabeth Groß, Felix Eckmann, Tassilo M. F. Restle, Thomas F. Fässler, Jonathan G. C. Veinot, and Bernhard Rieger**

Supporting Information

Surface-Anisotropic Janus Silicon Quantum Dots *via* Masking on Two-Dimensional Silicon Nanosheets

*Marc J. Kloberg, Haoyang Yu, Elisabeth Groß, Felix Eckmann, Tassilo M. F. Restle, Thomas F. Fässler, Jonathan G. C. Veinot, Bernhard Rieger**

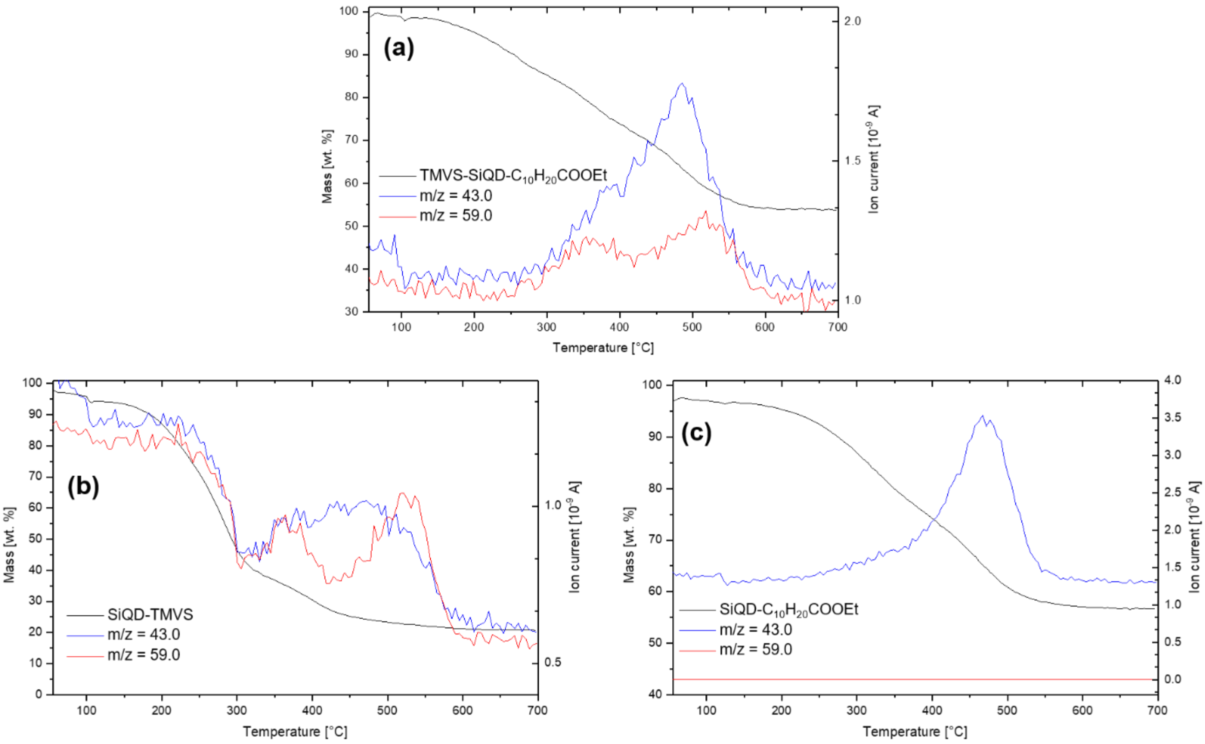

Figure S1. Thermogravimetric analysis – mass spectrometry (TGA-MS) spectra of (a) TMVS-SiQD-$C_{10}H_{20}$COOEt and the control samples (b) SiQD-TMVS and (c) SiQD-$C_{10}H_{20}$COOEt.

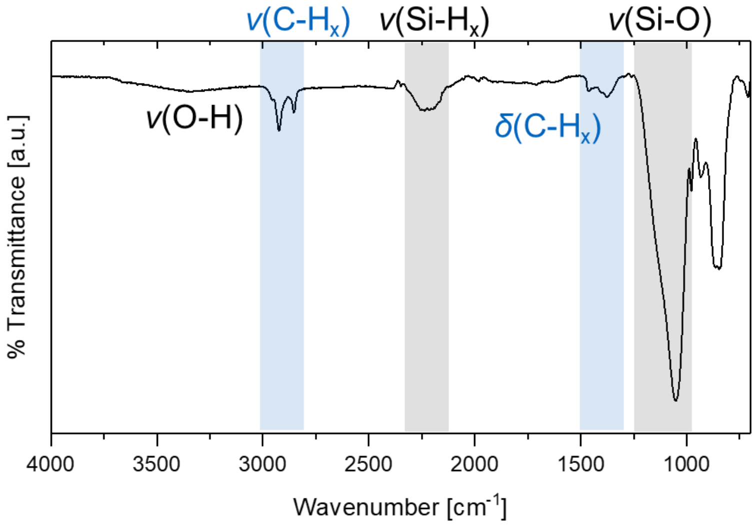

Figure S2. FT-IR spectrum of precipitate formed during the UV irradiation of TMVS-SiQD@SiNS-TMVS.

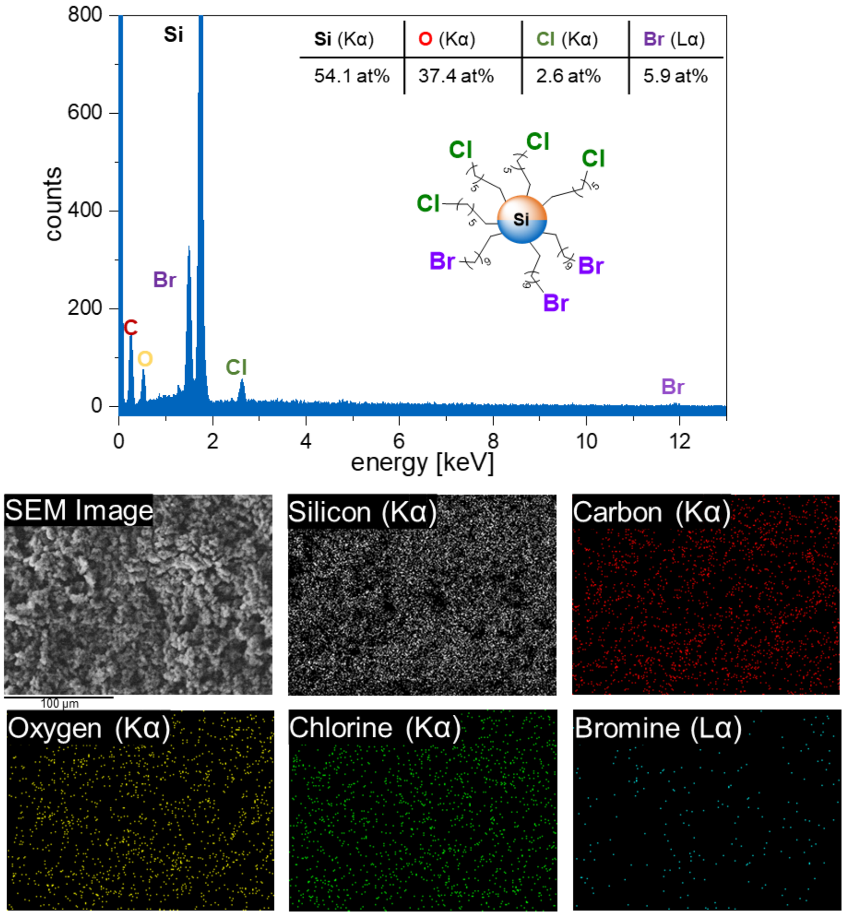

Figure S3. EDX spectrum of ClC_6H_{12}-SiQD-$C_{11}H_{22}Br$ with elemental composition and element mapping showing a homogeneous distribution throughout the sample.*

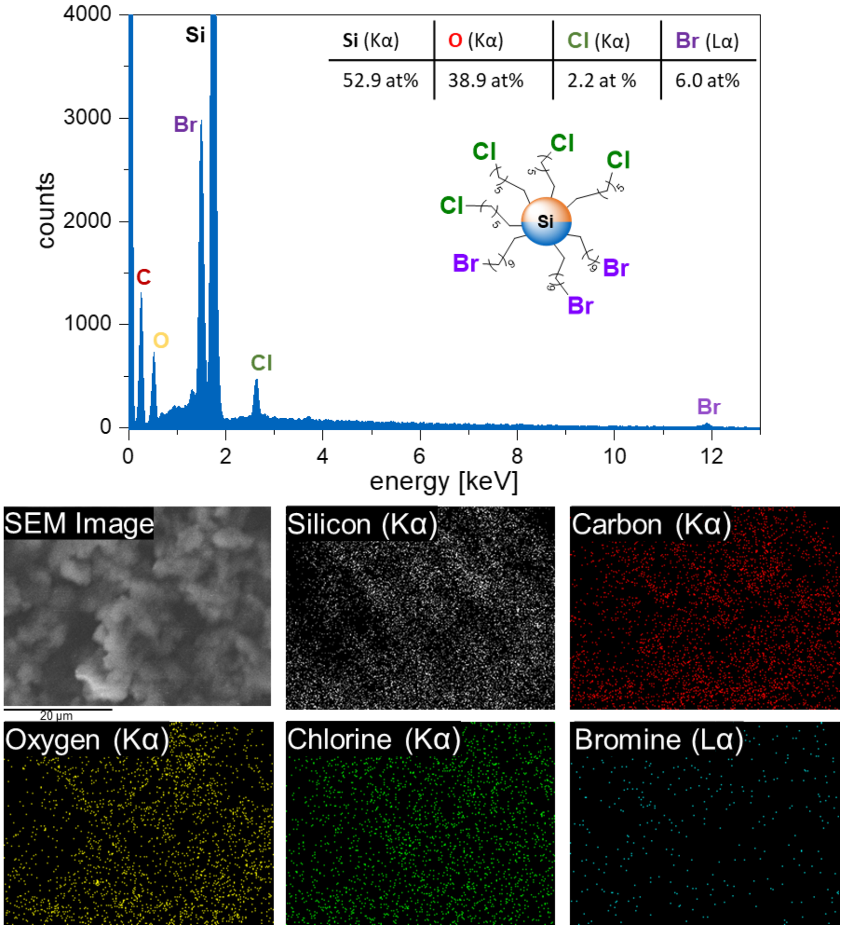

Figure S4. EDX spectrum of a second area of ClC_6H_{12}-SiQD-$C_{11}H_{22}Br$ with elemental composition and element mapping showing a homogeneous distribution throughout the sample.*

---

* The reported composition values are susceptible to strong inaccuracies due to matrix effects, sample preparation, hydrolysis of the halogens, and low relative content of halogens compared to Si, O, C. The values should be regarded as qualitative indicators.

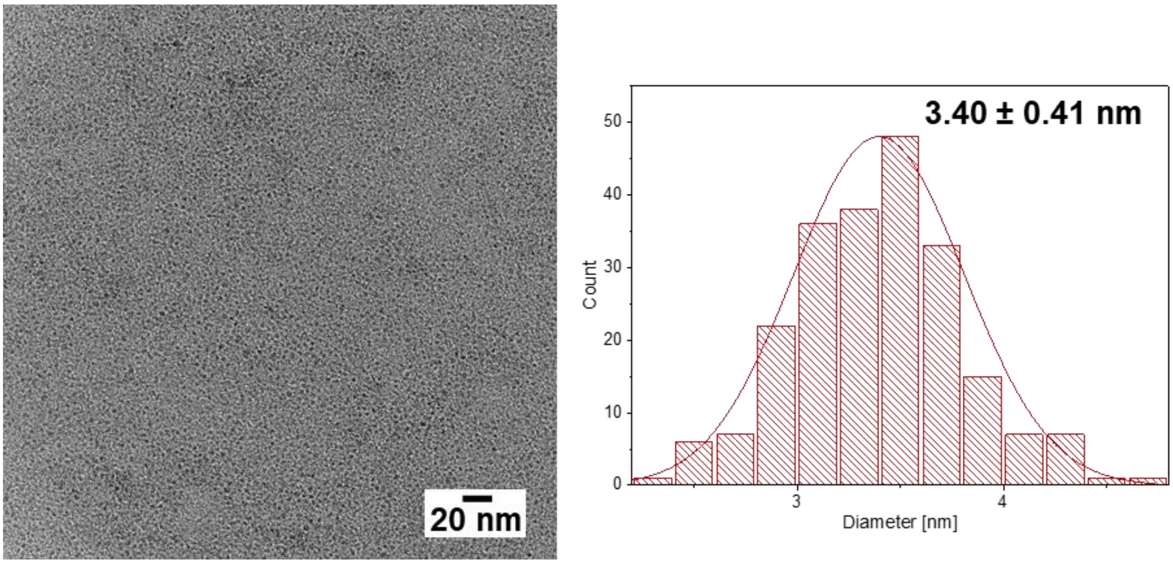

Figure S5. TEM image of TMVS-SiQD-$C_{10}H_{20}$COOEt and corresponding size distribution.

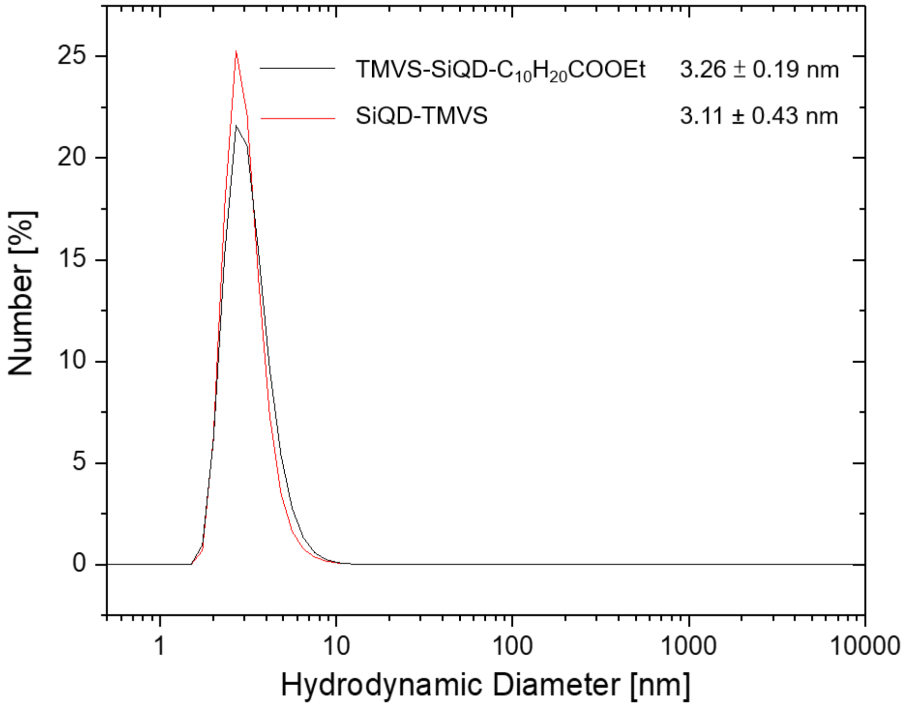

Figure S6. DLS measurements of TMVS-SiQD-$C_{10}H_{20}$COOEt and the control sample SiQD-TMVS.

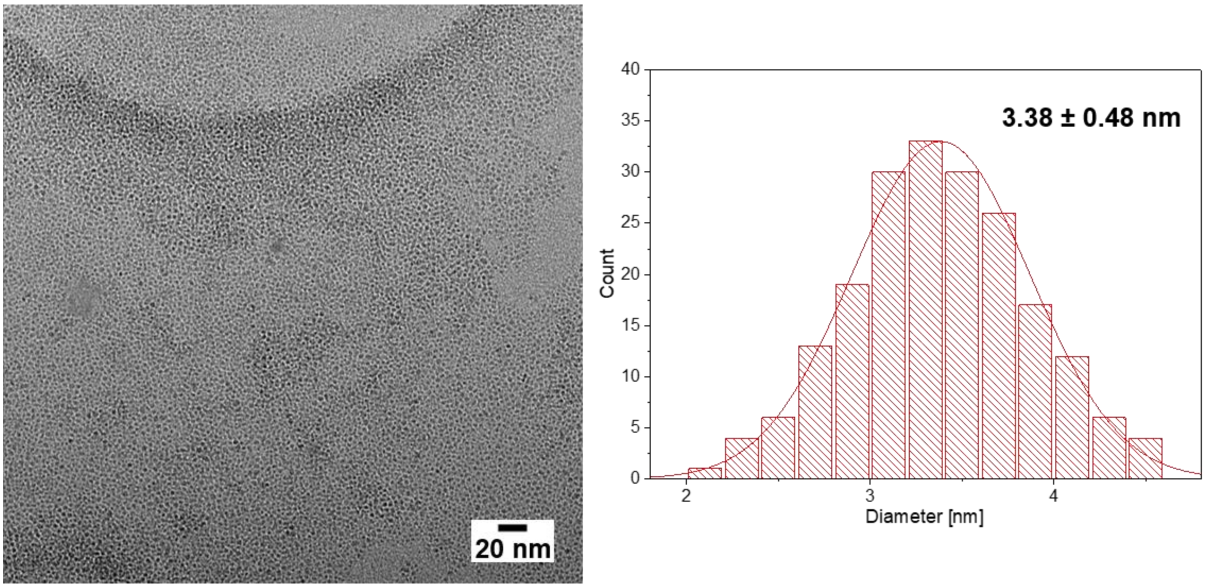

Figure S7. TEM image of control sample SiQD-TMVS and corresponding size distribution. The sample was synthesized from the same SiNC/SiO_2 composite and HF-acid etch as the previous Janus sample TMVS-SiQD-$C_{10}H_{20}$COOEt.

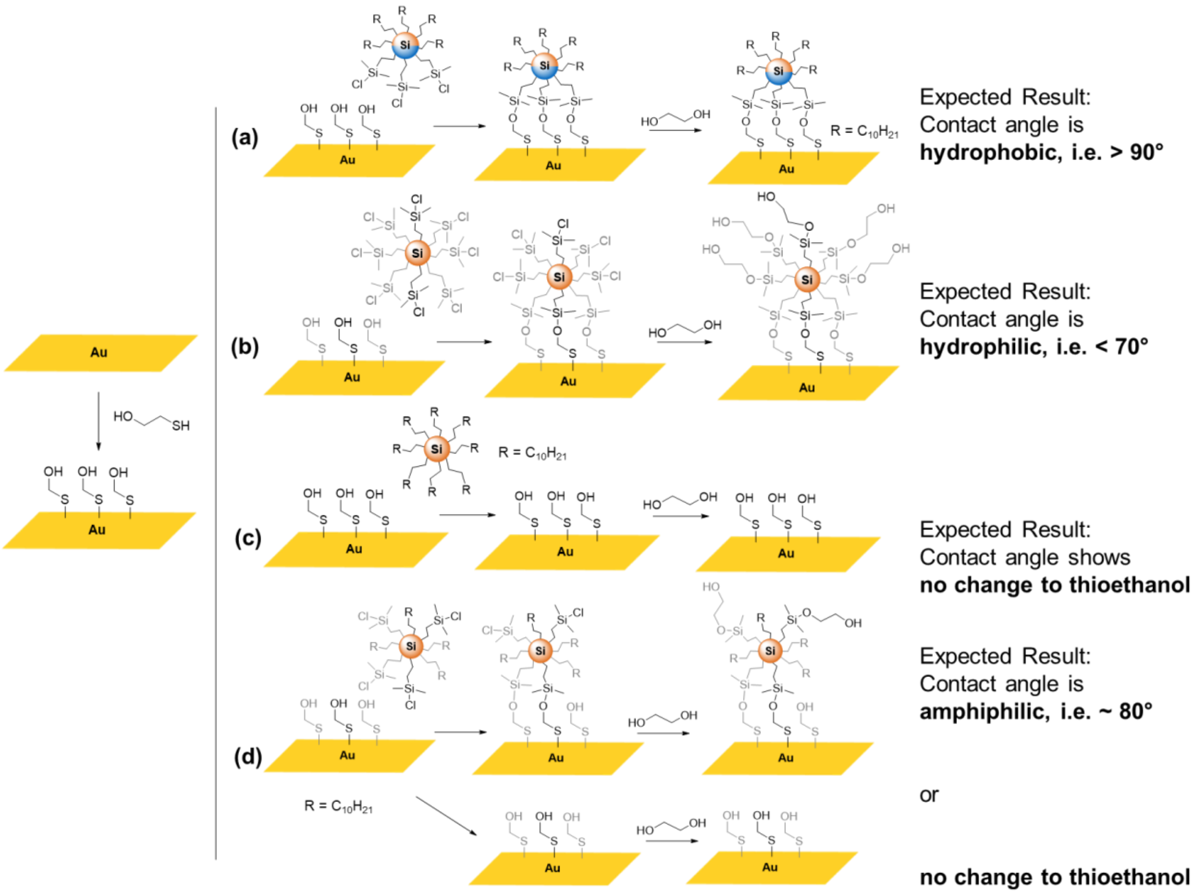

Figure S8. Synthetic strategy for the self-assembly of silicon nanocrystals on gold wafers and the expected contact angles. A gold wafer is functionalized with a monolayer of thioethanol and the silicon quantum dots are attached, followed by an ethylene glycol substitution step to render remaining chlorosilane groups hydrophilic. In order to keep conditions the same, this step is included in each sample. (a) Self-assembly of Janus SiQD ($H_{25}C_{12}$-SiQD-ClDMVS). (b) Self-assembly of isotropic chlorosilane SiQD (SiNC-ClDMVS). (c) Self-assembly of isotropic dodecyl SiQD (SiQD-$C_{12}H_{25}$). (d) Self-assembly of a mixed particle with chlorosilane and dodecyl groups (SiQD-$C_{12}H_{25}$/ClDMVS).

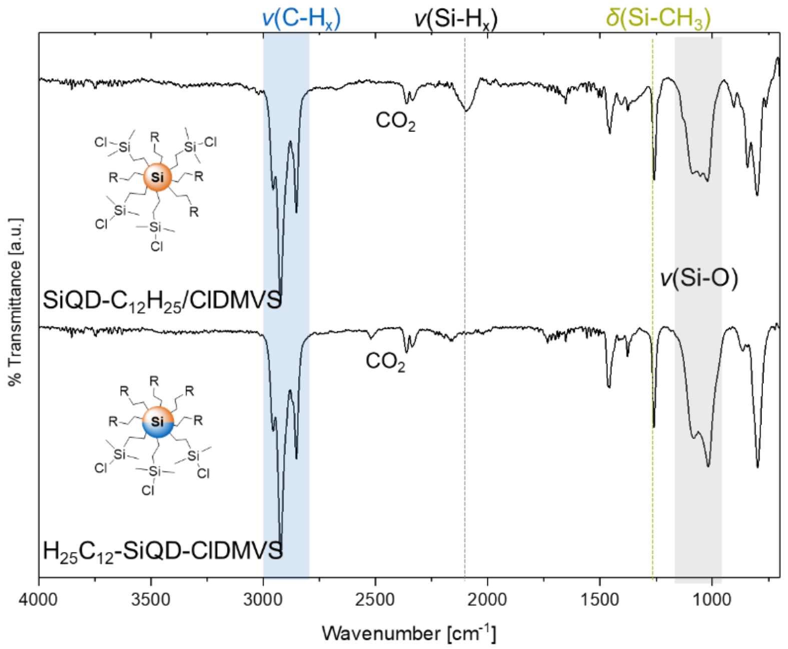

Figure S9. FT-IR spectra of a mixed-functionalized SiQD, SiQD-$C_{12}H_{25}$/ClDMVS (top), with the same functional groups as the Janus SiQD, $H_{25}C_{12}$-SiQD-ClDMVS (bottom).

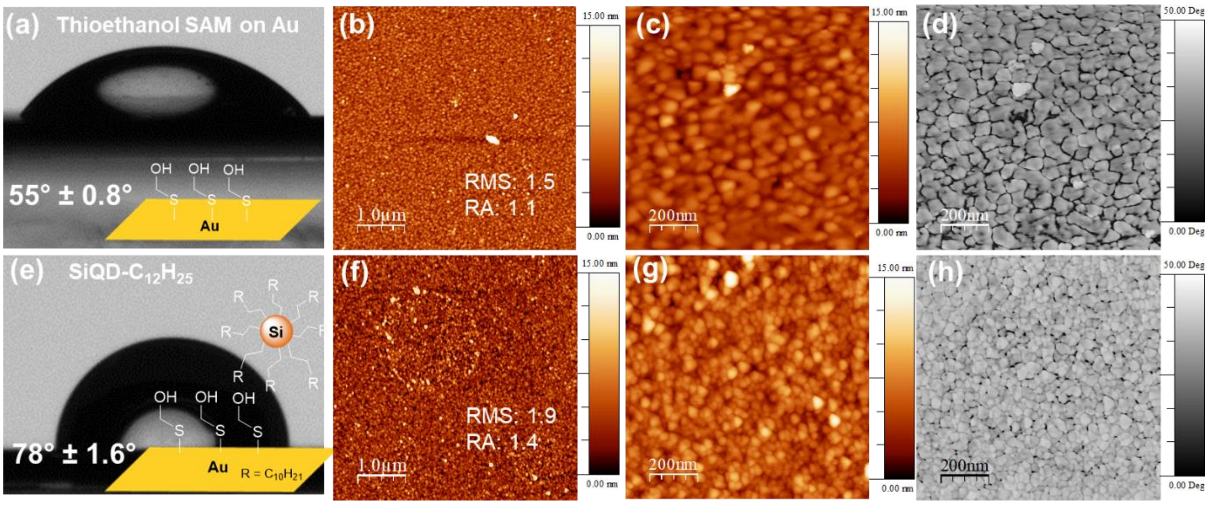

Figure S10. Control samples for the self-assembly of Janus SiQDs. Top row shows contact angle measurement (a), surface roughness (b) and AFM micrographs of the thioethanol reference wafer (b-d). The bottom row shows the contact angle (e), surface roughness (f) and AFM micrographs of SiQD-$C_{12}H_{25}$ deposition (f-h).

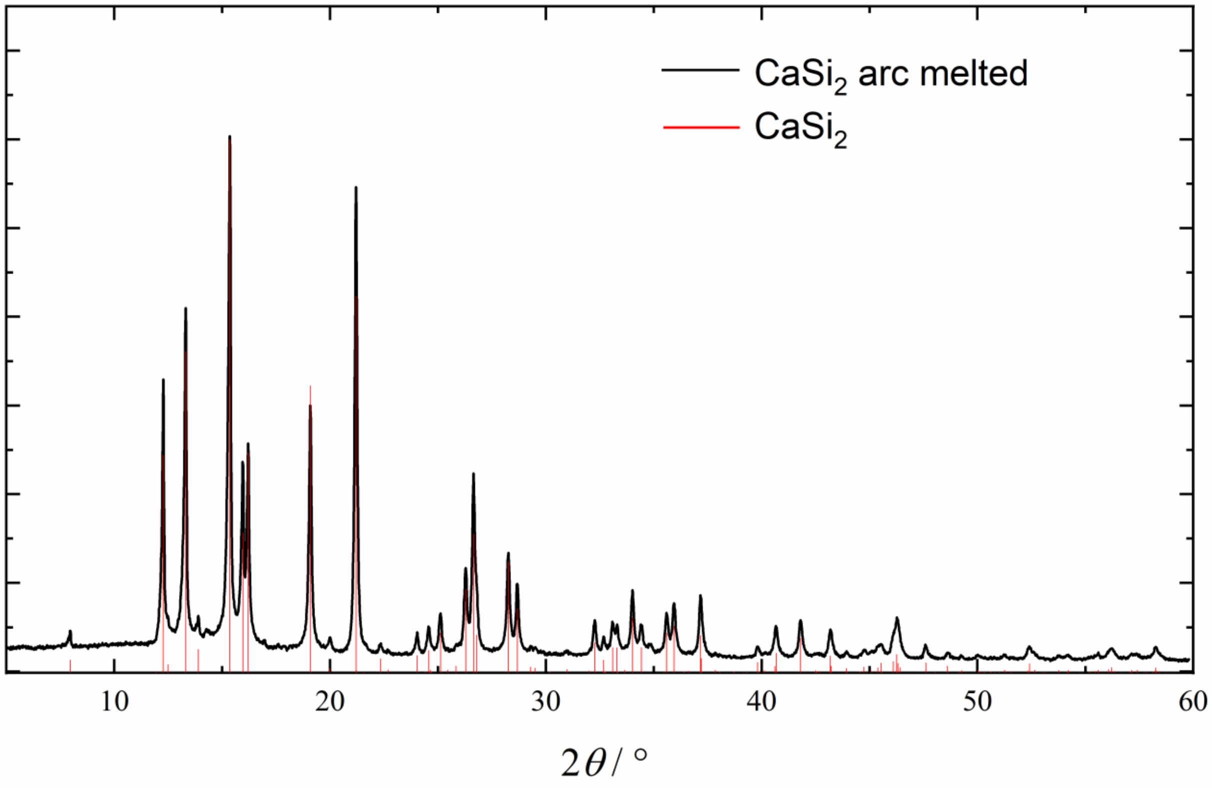

Figure S11: Experimental powder X-ray diffraction pattern of $CaSi_2$ synthesized via arc melting.

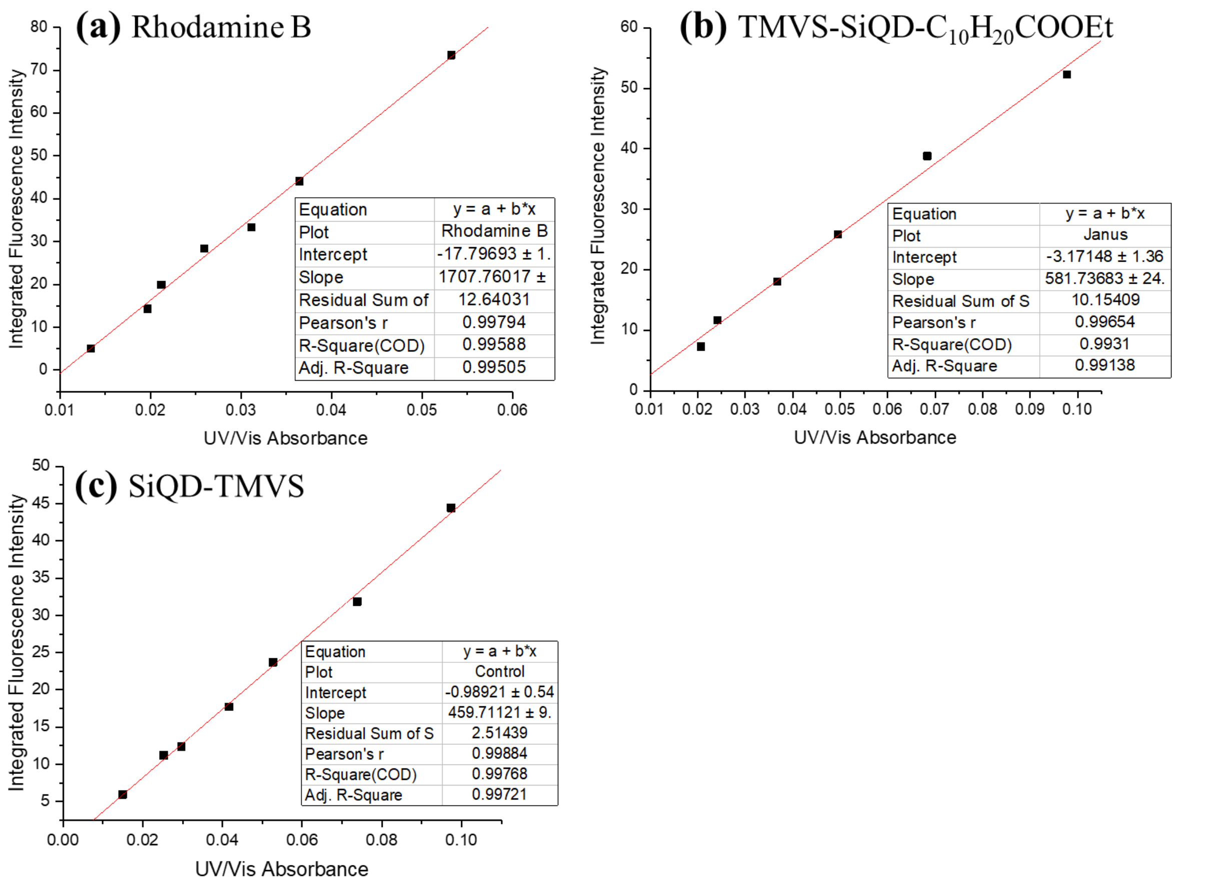

Figure S12: Fits and data used to calculate the photoluminescence quantum yield of Janus SiQDs and a SiQD control sample. Graphs represent the absorbance and the integrated area of fluorescence intensity at varying concentrations for (a) rhodamine B, (b) Janus SiQDs and (c) isotropic control sample.

Calculation of photoluminescence quantum yields using a known reference[1-3]

The photoluminescence quantum yield from a known reference is calculated using the following equation:

$$\Phi_S = \Phi_R \times \frac{F_S}{F_R} \times \frac{1-10^{-A_R}}{1-10^{-A_S}} \times \frac{n_S^2}{n_R^2} \qquad \text{eq.1}$$

Where Φ_S is the relative quantum yield, Φ_R is the quantum yield of a known reference (here 68% for rhodamine B in ethanol). F_x describes the integrated fluorescence intensity of sample

and reference, and A_x the absorbance at the excitation wavelength ($\lambda = 365\,nm$). n_x are the refractive indices of the solvents used (ethanol for rhodamine B and benzene for SiQDs).

For both sample and reference, the absorbance at the excitation wavelength and integrated fluorescence spectra were recorded under identical measurement conditions with varying concentrations. From the resulting linear fit of absorbance vs. integral area, the integrated fluorescence intensity is calculated for A = 0.05. The quantum yield is subsequently calculated from equation 1.

References

[1] S. P. Pujari, H. Driss, F. Bannani, B. van Lagen, H. Zuilhof, *Chem. Mater.* **2018**, *30*, 6503.

[2] A. M. Brouwer, *Pure and Applied Chemistry* **2011**, *83*, 2213.

[3] M. Grabolle, M. Spieles, V. Lesnyak, N. Gaponik, A. Eychmüller, U. Resch-Genger, *Anal. Chem.* **2009**, *81*, 6285.
